# Utility of Tablet-Based Eye Tracking for Early Screening of Poststroke Cognitive Impairment: Diagnostic Cohort Study

**DOI:** 10.2196/83194

**Published:** 2026-08-06

**Authors:** Boxu Xie, Ronghui Ren, Yuting Zhang, Xuan Wang, Junli Zhen

**Affiliations:** 1 Key Laboratory of Clinical Neurology, Ministry of Education, Hebei Medical University, 215 Heping West Road, Xinhua District, Shijiazhuang, Hebei, 050000, China, 86 1-383-112-9670; 2 Department of Neurology, the Second Hospital of Hebei Medical University, Shijiazhuang, Hebei, China; 3 Key Neurological Laboratory of Hebei Province, Shijiazhuang, Hebei, China

**Keywords:** acute ischemic stroke, poststroke cognitive impairment, eye tracking, tablet assessment, cognitive screening, mobile health technology

## Abstract

**Background:**

Poststroke cognitive impairment (PSCI) is a common and disabling complication after stroke; however, early screening remains challenging due to limited access to neuropsychological testing and the high cost of neuroimaging. Portable, tablet-based eye-tracking technology may offer a scalable, low-cost solution for early PSCI detection.

**Objective:**

This study aimed to evaluate the clinical utility of a tablet-based, AI-driven eye-tracking system for early screening of PSCI at 3 months after acute ischemic stroke. We sought to quantify oculomotor-cognitive associations and develop a practical nomogram for individualized risk prediction.

**Methods:**

We prospectively enrolled 142 hospitalized patients with acute cerebral infarction between May 2023 and October 2024, of whom 122 completed the 3-month follow-up and were included in the final analysis, along with 20 healthy community-dwelling controls. All patients underwent tablet-based eye tracking (visual paired comparison and antisaccade tasks) during the acute phase, as well as baseline and 3-month neuropsychological assessments. PSCI was defined using validated cutoffs. Multivariable logistic regression was used to identify independent predictors, and a nomogram was constructed. Internal validation was performed using bootstrap resampling (1000 samples).

**Results:**

At 3 months, out of 122 patients, 47 (38.5%) met PSCI criteria. Compared with patients with non-PSCI (n=75), patients with PSCI showed significantly prolonged correct saccade latency (median 322.96, IQR 209.45-445.59 ms vs 194.55, IQR 141.50-299.75 ms; *Z*=−4.03, *P*<.001), increased uncorrected error rate (median 30.00%, IQR 15.00%-42.00% vs 5.00%, IQR 0.00%-28.00%; *Z*=−4.24, *P*<.001), and reduced novelty preference ratio (median 1.44, IQR 0.97-1.70 vs 2.12, IQR 1.27-4.56; *Z*=−3.44, *P*=.001). Multivariable analysis identified 4 independent predictors of 3-month PSCI: older age (odds ratio [OR] 1.067 per year, 95% CI 1.009‐1.129; *P*=.02), lower education level (OR 0.841 per year, 95% CI 0.708‐0.999; *P*=.049), higher NIHSS (National Institutes of Health Stroke Scale) scores (OR 1.557 per point, 95% CI 1.075‐2.256; *P*=.02), and prolonged correct saccade latency (OR 1.004 per ms, 95% CI 1.000‐1.007; *P*=.04). A nomogram incorporating these 4 factors achieved good discriminative performance (area under the receiver operating characteristic curve 0.86, 95% CI 0.793‐0.927) with satisfactory calibration.

**Conclusions:**

Age, education, admission NIHSS, and correct saccade latency were identified as possible independent predictors of 3-month PSCI in this cohort. The tablet-based eye-tracking system, when combined with clinical variables, may represent a feasible approach for early PSCI screening. A nomogram based on these variables demonstrated high accuracy and potential clinical utility for early PSCI identification. This approach may facilitate early identification of high-risk patients and enable timely, personalized interventions in resource-limited settings.

## Introduction

Globally, stroke ranks as the second leading cause of mortality and frequently induces lifelong disabilities, including motor, cognitive, linguistic, and psychological impairments [1]. Among these sequelae, poststroke cognitive impairment (PSCI) constitutes a prevalent and debilitating neurological complication. PSCI is clinically defined as a syndrome fulfilling diagnostic criteria for cognitive impairment manifesting within 6 months post stroke. Formal neuropsychological assessment at 3 to 6 months post stroke is typically required for diagnosis [2]. This condition manifests clinically as deficits in memory, language comprehension, perceptual processing, visuospatial reasoning, and executive functioning [3]. Despite advances in acute stroke management, PSCI persists as a highly prevalent and debilitating condition [4], associated with elevated 5-year mortality and depression risks [5], thereby substantially contributing to long-term disability and reduced quality of life [6].

Early identification and management of PSCI represent critical clinical priorities [7]. Conventional tools such as the Mini-Mental State Examination (MMSE) and Montreal Cognitive Assessment (MoCA) exhibit limitations, including susceptibility to age, education, and emotional confounders, alongside significant time and resource requirements [8]. In recent years, mobile health (mHealth) technologies have shown great potential in enhancing the accessibility and scalability of medical assessments [9-11]. The integration of eye tracking into portable tablet devices allows for standardized, low-cost cognitive screening outside traditional clinical settings [12,13]. This approach aligns with growing emphasis on digital biomarkers and decentralized health care models, particularly in neurology and rehabilitation [14].

AI technology has gained substantial research interest owing to its objectivity and efficiency. AI-enhanced eye tracking quantitatively records ocular movements and gaze positions temporally and across task conditions. For target image projection and maintenance on the retinal fovea, coordinated eye movements are essential, assessed through saccadic, smooth pursuit, fixation, and visual search tasks [15]. As a noninvasive technique, eye tracking can identify cognitive impairment, monitor disease progression, and elucidate underlying cognitive processes [16]. Evidence suggests visual processing deficits may precede memory impairment in mild cognitive impairment (MCI), manifesting in altered eye movement patterns [17,18]. Patients with MCI frequently demonstrate elevated saccadic error rates, prolonged latency, and inverse correlations between erroneous saccade frequency and neuropsychological test performance [19]. In smooth pursuit tasks, patients with Alzheimer disease (AD) exhibit increased pursuit latency, reduced acceleration and gain, and frequent compensatory saccades correlating with cognitive decline [20].

These oculomotor abnormalities are not random; they directly reflect the functional state of the underlying neural circuits [21]. Precision eye tracking enables real-time parameter quantification, facilitating objective cognitive assessment [22]. Thus, AI-enhanced eye tracking captures millisecond-level ocular dynamics, offering an objective, quantifiable methodology for cognitive impairment evaluation.

Building on this evidence, researchers have recently extended eye tracking to stroke populations. Recent studies have applied eye tracking in stroke populations for various purposes, including augmenting stroke diagnosis [23], recognizing stroke through free viewing of faces [24], assessing the impact of poststroke fatigue on saccadic control [25], and evaluating memory-guided saccades in patients with subacute and chronic stroke [26]. These works confirm the feasibility of eye tracking in stroke-related conditions; however, none address early cognitive impairment. Regarding PSCI prediction, Zhou et al [27] built a nomogram using clinical and inflammatory markers. However, that model did not include oculomotor biomarkers. More recently, Chen et al [28] applied AI-assisted eye tracking with gait measurements to detect cognitive impairment in cerebral small vessel disease, directly supporting the utility of AI-driven oculomotor assessment in cerebrovascular disorders. A systematic review has further confirmed the diagnostic potential of eye tracking for AD and MCI, strengthening the rationale for applying oculomotor paradigms to cognitive screening [29]. Similarly, Maldonado-Díaz et al [30] used eye tracking to assess visual attention during virtual reality balance training in older adults with mild to moderate cognitive impairment, further illustrating the applicability of oculomotor measures across different cognitive impairment severities.

Despite these advances, no study has yet integrated a tablet-based, AI-enhanced eye-tracking system into a practical nomogram specifically for early PSCI screening. This gap motivated this study.

This study uses a noninvasive eye-tracking technology developed by Neuro Weave Ltd, which utilizes standard smartphone or tablet cameras to track subtle dynamic eye movements. By performing visual paired comparisons (VPCs) and antisaccade tasks on a tablet device, the assessment can be completed within minutes, significantly reducing time and labor compared to traditional paper-based scales. A study using this technology in patients with AD demonstrated a sensitivity of 82% and specificity of 91%, indicating high discriminative ability and low misdiagnosis risk in screening for AD [12]. Similarly, using the same tablet-based AI-driven eye-tracking technology, Wei et al [13] validated its performance in a large community-based cohort in China, achieving an area under the receiver operating characteristic curve (AUC) of 0.80 for detecting cognitive impairment and a high negative predictive value (87.2%), supporting its feasibility for population-level screening in resource-limited settings.

In summary, early warning and risk identification for PSCI are crucial. This study aims to explore the clinical utility of AI-based eye-tracking technology implemented on a tablet platform for screening early PSCI. By analyzing the relationship between eye movement characteristics and cognitive function in patients with stroke, we seek to develop and validate an AI eye-tracking model for early PSCI screening. The study hopes to provide an economical, convenient, and objective auxiliary diagnostic tool for early PSCI detection, thereby supporting clinical intervention and rehabilitation strategies to improve long-term prognosis and quality of life for patients with stroke.

## Methods

### Participants

We prospectively recruited 142 patients with acute cerebral infarction who were admitted to the Department of Neurology at the Second Hospital of Hebei Medical University between May 2023 and October 2024. Of these, 122 completed the 3-month follow-up assessments and were included in the final analysis, along with 20 healthy community-dwelling controls. Inclusion criteria for patients with stroke were as follows: (1) age 18 to 80 years; (2) within 14 days after symptom onset; (3) fulfilling the American Heart Association/American Stroke Association diagnostic criteria for stroke [31]; (4) capacity to undergo neuropsychological testing, physical examination, and brain magnetic resonance imaging (MRI); (5) corrected visual acuity sufficient for testing; and (6) provision of informed consent. Exclusion criteria included the following: (1) significant aphasia; (2) pregnancy, active malignancy, major organ failure, or life expectancy less than 1 year; (3) prestroke dementia or neurodegenerative disorders (eg, Lewy body dementia, AD, or secondary dementias); (4) Informant Questionnaire on Cognitive Decline in the Elderly score ≥4; and (5) congenital intellectual disability or severe neuropsychiatric conditions. Control participants were recruited using the following criteria: (1) age 18 to 80 years, (2) capacity to undergo neuropsychological testing, (3) corrected visual acuity sufficient for testing, and (4) provision of informed consent. Exclusion criteria included the following: (1) history of cognitive disorders, congenital intellectual disability, or other severe neuropsychiatric comorbidities; (2) history of stroke; or (3) current cognitive impairment defined as below-threshold scores on either MMSE or MoCA. Potentially eligible participants were identified based on admission records and clinical diagnosis. A purposive sampling strategy was used.

### Ethical Considerations

This study was approved by the Research Ethics Committee of the Second Hospital of Hebei Medical University (reference number 2023-C023) on March 31, 2023. All procedures followed the Declaration of Helsinki. Before enrollment, the researchers explained the study objectives in detail to the participants and their legal guardians, and written informed consent was obtained from the participants themselves or their guardians. All participants were informed that they could withdraw from the study at any time without penalty. Participant data were anonymized and deidentified before analysis; no personal identifiers are reported. No compensation was provided to participants for their involvement in this study.

### Neuropsychological Assessment

All participants completed cognitive and mood assessments, including the MMSE, MoCA, Self-Rating Anxiety Scale, and Self-Rating Depression Scale. Patients with stroke underwent repeated assessments at 3 months post stroke. PSCI diagnosis required meeting either criterion: MMSE score <17 (illiterate), <20 (1‐6 y education), or <24 (≥7 y education) or MoCA score <22 (validated cutoff demonstrating optimal sensitivity/specificity) [32,33]. Patients were stratified into PSCI and non-PSCI (NPSCI) subgroups based on 3-month cognitive performance. All assessments were administered by neurologists certified in standardized neuropsychological testing protocols. The neurologists who administered the 3-month neuropsychological assessments were blinded to the patients’ baseline eye-tracking results and the initial clinical assessments.

### Eye-Tracking Technology Task

**Figure 1.**
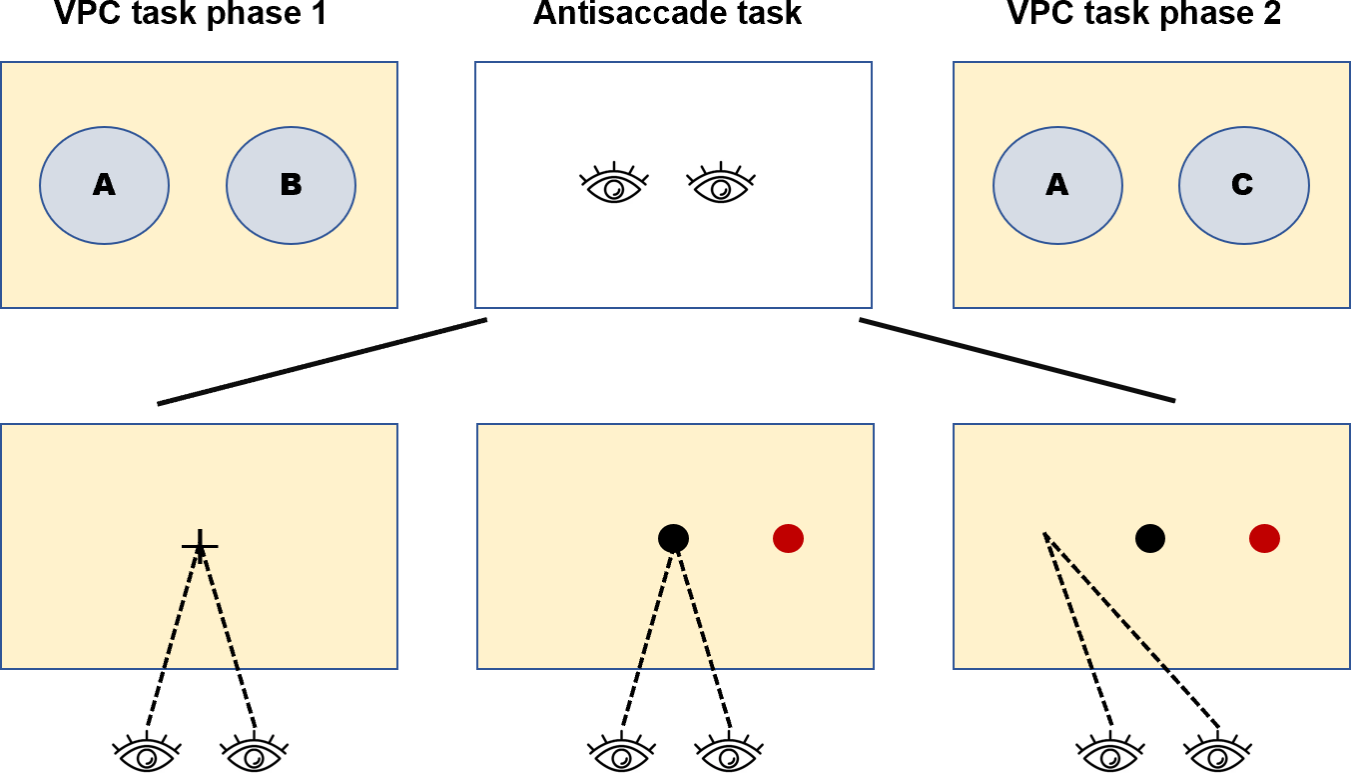
The visual paired comparisons (VPC) and antisaccade task.

Eye-tracking data were acquired during the acute phase of cerebral infarction using a Xiaomi Pad 5 Pro 12.4 Android tablet (12.4-inch display, 2560×1600 resolution, 60 Hz refresh rate) with a front-facing camera capable of recording 1080p video at 30 frames per second, running a proprietary research software developed by Shanghai Neuroweave Technology Co, Ltd, for Android 8.0+ devices. This software is a research tool rather than a commercial retail product. Its validity for cognitive screening has been demonstrated in previous studies [12,13]. The system initiated recording upon task start, transmitting videos to Alibaba Cloud for processing via an AI eye-tracking model that integrated computer vision and deep learning architectures for facial landmark detection, head pose estimation, pupil tracking, and gaze vector calculation. Demographic data were input preassessment via user interface, followed by head pose calibration requiring participants to position within an on-screen virtual frame to standardize distance and minimize motion artifacts. The 6-minute protocol comprised (1) pretask calibration optimizing device-specific tracking, (2) VPC phase 1: five 5-second image sets for memorization, (3) antisaccade task (~3 min): central fixation with rapid gaze shifts to contralateral space upon peripheral red dot appearance, and (4) VPC phase 2: ten 6-second sets (1 familiar/1 novel image per set) where participants viewed novel stimuli while camera-recorded videos underwent cloud-based processing to extract quantitative eye movement-related parameters. After processing the video, a series of eye movement–related parameters were established, as shown in Table 1. An example diagram of the eye-tracking task is shown in Figure 1.

**Table 1.** Oculomotor parameters quantified during eye-tracking assessment.

Parameter	Definition	Unit/formula
Antisaccade task		
Latency	Time delay for saccade initiation after target onset	ms
Correct saccade latency	Latency of the first directionally accurate saccade	ms
Error rate	Percentage of initial saccades with incorrect direction	%
Peak velocity (horizontal)	Maximum horizontal velocity during saccades	Pixels/ms
Correction latency	Delay in generating corrective saccades after erroneous initial saccades	ms
Uncorrected error rate	Percentage of trials where corrective saccades remained directionally inaccurate	%
Corrected saccade latency	Latency of the first accurate saccade post correction	ms
Presaccadic fixation count	Number of fixation events before target onset	Count
Postsaccadic fixation count	Number of fixation events after target onset	Count
Fixation stability	Number of fixation clusters prior to target appearance	Count
Blinks during fixation	Fixation events contaminated by blinks	Count
Initial saccadic gain	Horizontal displacement amplitude of the first saccade	Pixels
Terminal saccadic gain ratio	Ratio of horizontal distance: Δ (first saccade end point → initial fixation)/Ref (final presaccade fixation → target)	Δ/Ref (unitless)
VPC^a^ task		
Novelty preference ratio	Dwell time ratio (novel image/familiar image)	Ratio (unitless)
Total fixation duration	Summed duration of all fixation events	ms
Fixation events with blinks	Fixation epochs interrupted by blinks	Count

a VPC: visual paired comparisons.

All tests were conducted in an inpatient setting, in a dedicated examination room to ensure a quiet and controlled environment. No other patients were present during testing. Patients remained seated during the test. The tablet was mounted on a stand and placed on a desk at a comfortable viewing distance. A single investigator who had undergone standardized protocol training administered the test. Before the test started, the investigator used a pre-prepared script to explain the procedure to the patient. Once the test began, the software provided voice guidance (on-screen text and narration) to instruct the patient through each step.

Head pose calibration required the patient to align their face within an on-screen virtual frame. The adjustment process typically took about 3 seconds; once the patient’s head was correctly positioned, calibration was completed immediately. If the initial alignment failed, the investigator gently reminded the patient to stay still, and the patient readjusted within another few seconds. This process did not cause noticeable fatigue, even in patients with hand tremors. The complete testing procedure (including calibration, VPC, and antisaccade tasks) took approximately 6 minutes per participant, which was well tolerated.

### Statistical Analysis

As the index test comprised multiple continuous oculomotor parameters, no single test positivity cutoff was predefined. Instead, a multivariable logistic regression model was constructed to combine these metrics with clinical variables for PSCI risk prediction. The model’s performance was evaluated using AUC, sensitivity, and specificity at the optimal probability threshold derived from the receiver operating characteristic curve. All statistical analyses were performed using IBM SPSS 27.0 and R software (v4.2.2; R Foundation for Statistical Computing). The sample size was determined based on feasibility (all eligible patients enrolled during the study period); no formal sample size calculation was performed. Continuous variables with normal distribution were expressed as mean (SD) and compared using independent *t* tests or one-way ANOVA. Nonnormally distributed continuous variables were reported as median (IQR) and analyzed using Kruskal-Wallis *H* tests or Mann-Whitney *U* tests. Categorical variables were described as frequencies (%) and compared via chi-square tests or Fisher exact tests. Variables with *P*<.05 in univariate logistic regression analyses for PSCI were incorporated into multivariate binary logistic regression using stepwise selection to construct a PSCI risk prediction model. Model performance was validated internally. Statistical significance was defined as *P*<.05. Missing data on the 3-month follow-up were handled by complete case analysis; only patients with complete data were included in the final analysis.

## Results

### General Clinical Data Analysis of the Patients

**Figure 2.**
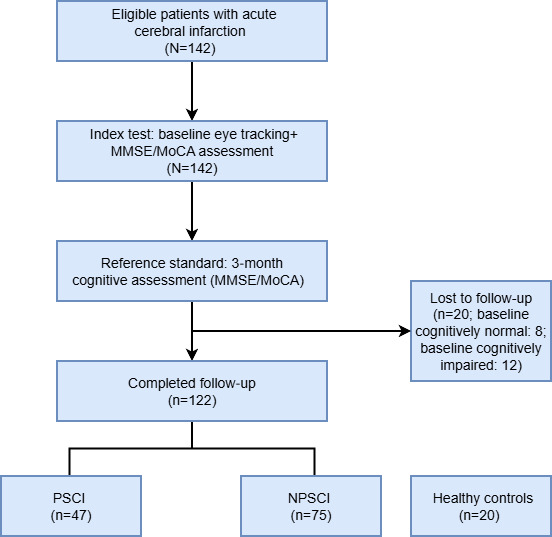
STARD (Standards for Reporting Diagnostic Accuracy Studies) flow diagram. MMSE: Mini-Mental State Examination; MoCA: Montreal Cognitive Assessment; NPSCI: non–poststroke cognitive impairment; PSCI: poststroke cognitive impairment.

This study enrolled 142 patients with acute cerebral infarction who met the inclusion/exclusion criteria, all of whom underwent baseline neuropsychological assessments during hospitalization. Of these, 122 completed 3-month follow-up evaluations, comprising the study cohort. PSCI developed in 47 patients (38.5% incidence), with 75 classified as NPSCI (Figure 2; see Checklist 1). Compared to patients with NPSCI, the PSCI group had a significantly higher proportion of females (18/47, 38% vs 14/75, 19%; χ²_1_=5.76, *P*=.02), older age (mean 64.23, SD 8.95 vs mean 56.68, SD 10.42; *t*_120_*=*−4.11, *P*<.001), lower education level (6.00, IQR 6.00‐9.00 vs 9.00, IQR 6.00‐12.00; *Z=*−3.68, *P*<.001), and elevated admission neurological severity scores (NIHSS [National Institutes of Health Stroke Scale]=3.00, IQR 2.00‐6.00 vs 2.00, IQR 1.00‐3.00; *Z=*−3.68, *P*<.001; mRS [Modified Rankin Scale]=2.00, IQR 1.00‐4.00 vs 1.00, IQR 1.00‐3.00; *Z=*−3.06, *P*=.002), whereas no significant differences existed in BMI, history of prior stroke, hypertension, diabetes, coronary artery disease, smoking, alcohol use, or anxiety/depression prevalence (Table 2).

**Table 2.** Comparative analysis of demographic and clinical characteristics across study groups.

Variable	PSCI^a^ (n=47)	NPSCI^b^ (n=75)	HC^c^ (n=20)	PSCI vs NPSCI		NPSCI vs HC	
				*t/Z/*χ^2^ (*df*)	*P* value	*t/Z/*χ^2^ (*df*)	*P* value
Age (y)	64.23 (8.95)^d^	56.68 (10.42)^d^	61.00 (44.75-62.75)^e^	−4.11 (120)^f^	<.001	−0.17	.87
Education level (y)	6.00 (6.00-9.00)^e^	9.00 (6.00-12.00)^e^	9.00 (6.75-12.00)^e^	−3.68	<.001	−0.44	.66
BMI (kg/m^2^)	25.06 (23.31-27.12)^e^	25.96 (23.94-27.92)^e^	25.00 (2.76)^d^	−1.33	.18	1.52	.13
NIHSS^g^	3.00 (2.00-6.00)^e^	2.00 (1.00-3.00)^e^	—^h^	−3.68	<.001	—	—
mRS^i^	2.00 (1.00-4.00)^e^	1.00 (1.00-3.00)^e^	—	−3.06	.002	—	—
Baseline MMSE^j^	21.00 (17.00-25.00)^e^	27.00 (25.00-29.00)^e^	28.50 (28.00-30.00)^e^	−6.49	<.001	−1.63	.10
Baseline MoCA^k^	15.00 (12.00-19.00)^e^	24.00 (20.00-26.00)^e^	26.00 (26.00-27.00)^e^	−7.47	<.001	−2.45	.01
Follow-up MMSE	22.00 (17.00-24.00)^e^	28.00 (27.00-29.00)^e^	—	−8.40	<.001	—	—
Follow-up MoCA	16.00 (12.00-20.00)^e^	25.00 (23.00-27.00)^e^	—	−9.18	<.001	—	—
Sex, n (%)				5.76 (1)^l^	.02	10.75 (1)^l^	.001
Male	29 (62)	61 (81)	9 (45)	—	—	—	—
Female	18 (38)	14 (19)	11 (55)	—	—	—	—
History of stroke, n (%)	15 (32)	13 (17)	—	3.47 (1)^l^	.06	—	—
Hypertension, n (%)	32 (68)	49 (65)	9 (45)	0.10 (1)^l^	.75	2.75 (1)^l^	.10
Diabetes, n (%)	10 (21)	17 (23)	3 (15)	0.03 (1)^l^	.86	0.02 (1)^l^	.66
Coronary artery disease, n (%)	1 (2)	3 (4)	1 (5)	—	>.99	—	>.99
Smoking, n (%)	15 (32)	32 (43)	6 (30)	1.41 (1)^l^	.24	0.89 (1)^l^	.36
Alcohol use, n (%)	12 (26)	23 (31)	7 (35)	0.37 (1)^l^	.54	0.14 (1)^l^	.71
Anxiety, n (%)	7 (15)	6 (8)	0 (0)	1.44 (1)^l^	.23	—	.34
Depression, n (%)	7 (15)	5 (7)	0 (0)	1.38 (1)^l^	.24	—	.58

a PSCI: poststroke cognitive impairment.

b NPSCI: non–poststroke cognitive impairment.

c HC: healthy control.

d Continuous variables with normal distribution were expressed as mean (SD).

e Nonnormally distributed continuous variables were reported as median (IQR).

f For continuous variables, independent-samples *t* tests were used for normally distributed data.

g NIHSS: National Institutes of Health Stroke Scale.

h Not applicable.

i mRS: Modified Rankin Scale.

j MMSE: Mini-Mental State Examination.

k MoCA: Montreal Cognitive Assessment.

l For categorical variables, χ2 tests were used to compare proportions between groups.

### Analysis of Eye-Tracking Metrics

**Figure 3.**
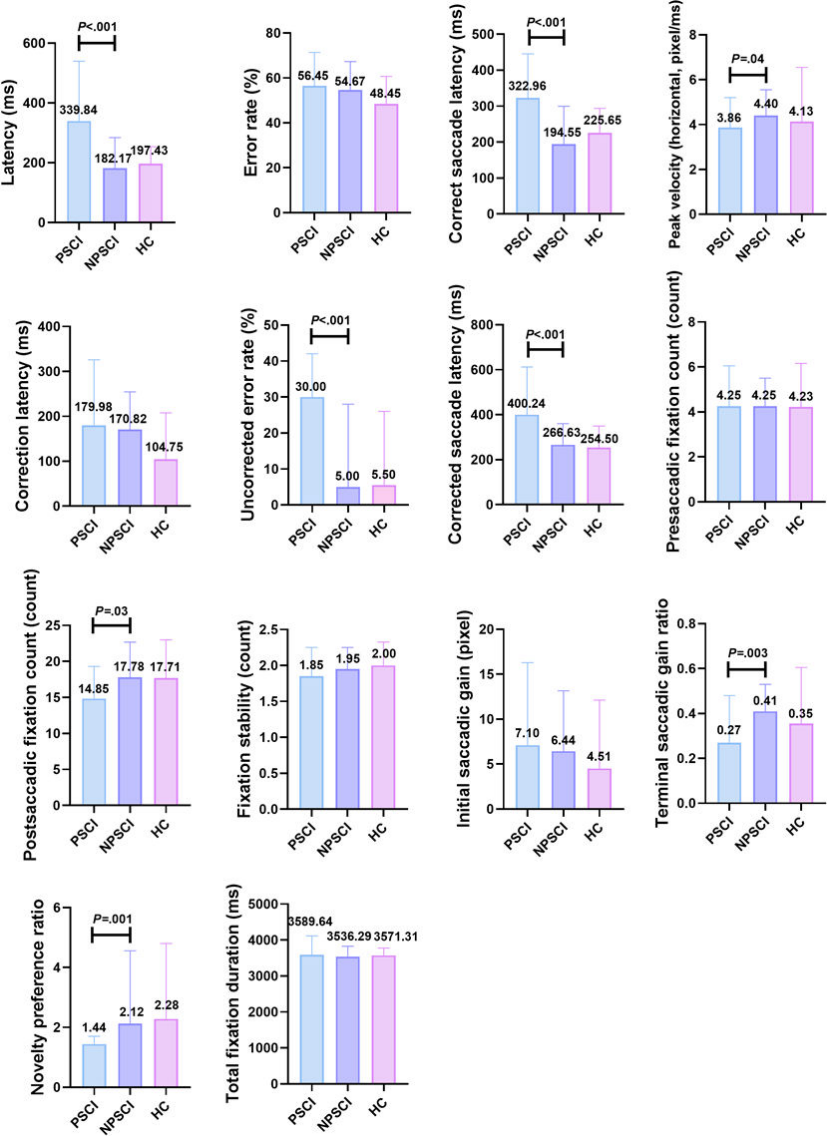
Comparative analysis of quantitative oculomotor metrics across study cohorts. HC: healthy control; NPSCI: non–poststroke cognitive impairment; PSCI: poststroke cognitive impairment.

Analysis of eye-tracking metrics revealed that patients with PSCI exhibited markedly prolonged saccadic processing, demonstrating significantly higher saccade latency (*Z*=−4.77, *P*<.001), correct saccade latency (*Z*=−4.03, *P*<.001), uncorrected error rate (*Z*=−4.24, *P*<.001), and corrected saccade latency (*Z*=−4.62, *P*<.001) compared to NPSCI controls. Conversely, the PSCI group showed significantly reduced oculomotor efficiency, characterized by lower peak horizontal velocity (*Z*=−2.01, *P*=.04), diminished postsaccadic fixation count (*Z*=−2.20, *P*=.03), impaired terminal saccadic gain ratio (*Z*=−2.97, *P*=.003), and decreased novelty preference ratio (*Z*=−3.44, *P*=.001). However, fundamental visuomotor integration remained intact between groups, with no significant differences in error rate, correction latency, presaccadic fixation count, fixation stability, blinks during fixation, initial saccadic gain, total fixation duration, or VPC fixation events with blinks (all *P*>.05; Figure 3; see Multimedia Appendix 1 for exact *P* values). These findings suggest that executive-attentional deficits in PSCI selectively disrupt higher-order saccadic control and novelty processing, while preserving basic oculomotor functions.

### Comparative Analysis of Demographic and Eye-Tracking Metrics Between Healthy Controls and NPSCI Groups

To investigate whether oculomotor performance in patients with NPSCI aligns with neurologically intact individuals, we compared quantitative eye-tracking metrics between 20 age-matched healthy control (HC) and the NPSCI cohort. Comprehensive analysis demonstrated no statistically significant differences in any eye-tracking parameters (all *P*>.05; Figure 3; see Multimedia Appendix 2 for exact *P* values), including saccade latency, novelty preference ratio, and fixation stability metrics, confirming that oculomotor function in patients with NPSCI remains within normative ranges despite cerebrovascular pathology. These findings suggest preserved visuomotor network integrity in patients living with stroke without cognitive impairment.

### Logistic Regression Analysis of Eye-Tracking Metrics in Patients With PSCI

Binary logistic regression analysis identified significant predictors of PSCI, with demographic factors including sex (male vs female, with female as the reference; OR 0.370, 95% CI 0.162‐0.845; *P*=.02), advanced age (OR 1.084, 95% CI 1.038‐1.132 per year; *P*<.001), and lower education level (OR 0.801, 95% CI 0.710‐0.904 per year; *P*<.001), alongside clinical indicators of higher admission NIHSS (OR 1.463, 95% CI 1.202‐1.781 per point; *P*<.001) and elevated mRS scores (OR 1.701, 95% CI 1.220‐2.373 per point; *P*=.002). Critically, oculomotor metrics independently predicted PSCI risk: prolonged saccade latency (OR 1.005, 95% CI 1.003‐1.007 per ms; *P*<.001), increased correct saccade latency (OR 1.004, 95% CI 1.002‐1.006 per ms; *P*=.001), elevated uncorrected error rate (OR 1.043, 95% CI 1.021‐1.066 per %; *P*<.001), extended corrected saccade latency (OR 1.005, 95% CI 1.003‐1.007 per ms; *P*<.001), reduced postsaccadic fixation count (OR 0.949, 95% CI 0.903‐0.998 per count; *P*=.04), and diminished novelty preference ratio (OR 0.686, 95% CI 0.532‐0.884 per unit; *P*=.004). These findings demonstrate that oculomotor dysfunction significantly contributes to PSCI pathogenesis beyond traditional clinical factors (complete models in Table 3).

**Table 3.** Univariate logistic regression analysis.

Variable	*B* (SE)	Wald	*P* value	OR^a^ (95% CI)
Sex (male vs female)^b^	−0.995 (0.422)	5.565	.02	0.370 (0.162-0.845)
Age (y)	0.081 (0.022)	13.432	<.001	1.084 (1.038-1.132)
Education level (y)	−0.222 (0.062)	12.953	<.001	0.801 (0.710-0.904)
NIHSS^c^	0.381 (0.100)	14.366	<.001	1.463 (1.202-1.781)
mRS^d^	0.531 (0.170)	9.799	.002	1.701 (1.220-2.373)
Latency (ms)	0.005 (0.001)	16.824	<.001	1.005 (1.003-1.007)
Correct saccade latency (ms)	0.004 (0.001)	11.192	.001	1.004 (1.002-1.006)
Uncorrected error rate (%)	0.042 (0.011)	15.076	<.001	1.043 (1.021-1.066)
Corrected saccade latency (ms)	0.005 (0.001)	16.014	<.001	1.005 (1.003-1.007)
Postsaccadic fixation count (count)	−0.052 (0.025)	4.222	.04	0.949 (0.903-0.998)
Novelty preference ratio	−0.377 (0.130)	8.464	.004	0.686 (0.532-0.884)

a OR: odds ratio.

b Sex was coded as male=1 and female=0; therefore, an OR<1 indicates a lower risk for males relative to females.

c NIHSS: National Institutes of Health Stroke Scale.

d mRS: Modified Rankin Scale.

Multivariate logistic regression analysis was performed after excluding variables with variance inflation factors greater than 5 to mitigate multicollinearity, retaining significant predictors from univariate screening. The final validated model demonstrated that advanced age (OR 1.067, 95% CI 1.009‐1.129 per year; *P*=.02), lower education level (OR 0.841, 95% CI 0.708‐0.999 per year; *P*=.049), higher NIHSS scores (OR 1.557, 95% CI 1.075‐2.256 per point; *P*=.02), and prolonged correct saccade latency (OR 1.004, 95% CI 1.000‐1.007 per ms; *P*=.04) independently predicted PSCI development. Clinically, each 1-year age increase elevates PSCI risk by 6.7% (OR 1.067), while each additional education year reduces risk by 15.9% (1/0.841≈1.189). A 1-point NIHSS increase confers 55.7% higher PSCI probability, and critically, every 100-ms increase in correct saccade latency nearly doubles PSCI likelihood (OR 1.49, 95% CI 1.00‐2.22, calculated as 1.004^100^). These results establish eye-tracking metrics as novel predictors complementing traditional stroke severity indices (complete models in Table 4).

**Table 4.** Multivariate logistic regression analysis.

Variable	*B* (SE)	Wald	*P* value	OR^a^ (95% CI)
Sex (male vs female)^b^	−0.383 (0.633)	0.366	.55	0.682 (0.197-2.357)
Age (y)	0.065 (0.029)	5.200	.02	1.067 (1.009-1.129)
Education level (y)	−0.173 (0.088)	3.867	.049	0.841 (0.708-0.999)
NIHSS^c^	0.443 (0.189)	5.482	.02	1.557 (1.075-2.256)
mRS^d^	−0.221 (0.349)	0.402	.53	0.801 (0.404-1.590)
Correct saccade latency (ms)	0.004 (0.002)	4.342	.04	1.004 (1.000-1.007)
Uncorrected error rate (%)	0.014 (0.015)	0.885	.35	1.014 (0.985-1.043)
Postsaccadic fixation count (count)	−0.023 (0.039)	0.357	.56	0.977 (0.905-1.054)
Novelty preference ratio	−0.023 (0.141)	0.027	.87	0.977 (0.741-1.289)
Constant	−4.451 (2.309)	3.717	.05	0.012

a OR: odds ratio.

b Sex was coded as male=1 and female=0; therefore, an OR<1 indicates a lower risk for males relative to females.

c NIHSS: National Institutes of Health Stroke Scale.

d mRS: Modified Rankin Scale.

### Development of a Multivariable Predictive Model for PSCI Incorporating a Risk Nomogram

**Figure 4.**
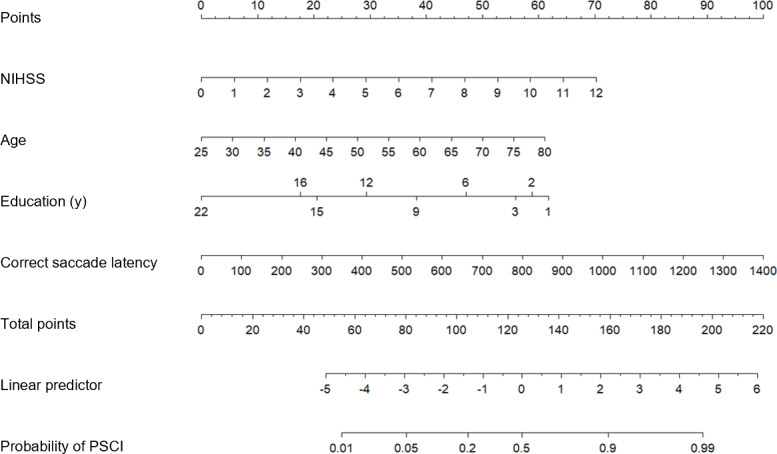
Nomogram model for predicting poststroke cognitive impairment (PSCI) risk. NIHSS: National Institutes of Health Stroke Scale.

Based on the multivariate logistic regression analysis, advanced age, lower education level, higher NIHSS scores, and prolonged correct saccade latency were identified as independent predictors of PSCI in patients with acute ischemic stroke. These factors were integrated into a clinically actionable point-based nomogram (Figure 4) for individualized PSCI risk estimation at 3 months post stroke. The nomogram operates through three sequential steps: (1) variable scoring: assign points for each predictor (age, education, NIHSS, and latency) using the top axis scale. (2) Total points calculation: sum all individual scores to determine aggregate risk points. (3) Probability conversion: map total points to the bottom probability axis, directly indicating PSCI likelihood. For example, a 70-year-old (50 points) with 6 years of education (35 points), NIHSS=4 (23 points), and correct saccade latency=500 ms (35 points) would have 143 total points, corresponding to 70% PSCI probability. This visual tool enables rapid risk stratification at bedside without computational tools, demonstrating how oculomotor metrics enhance traditional clinical prediction.

### Validation of the PSCI Risk Nomogram: Discriminatory Accuracy and Calibration Performance

**Figure 5.**
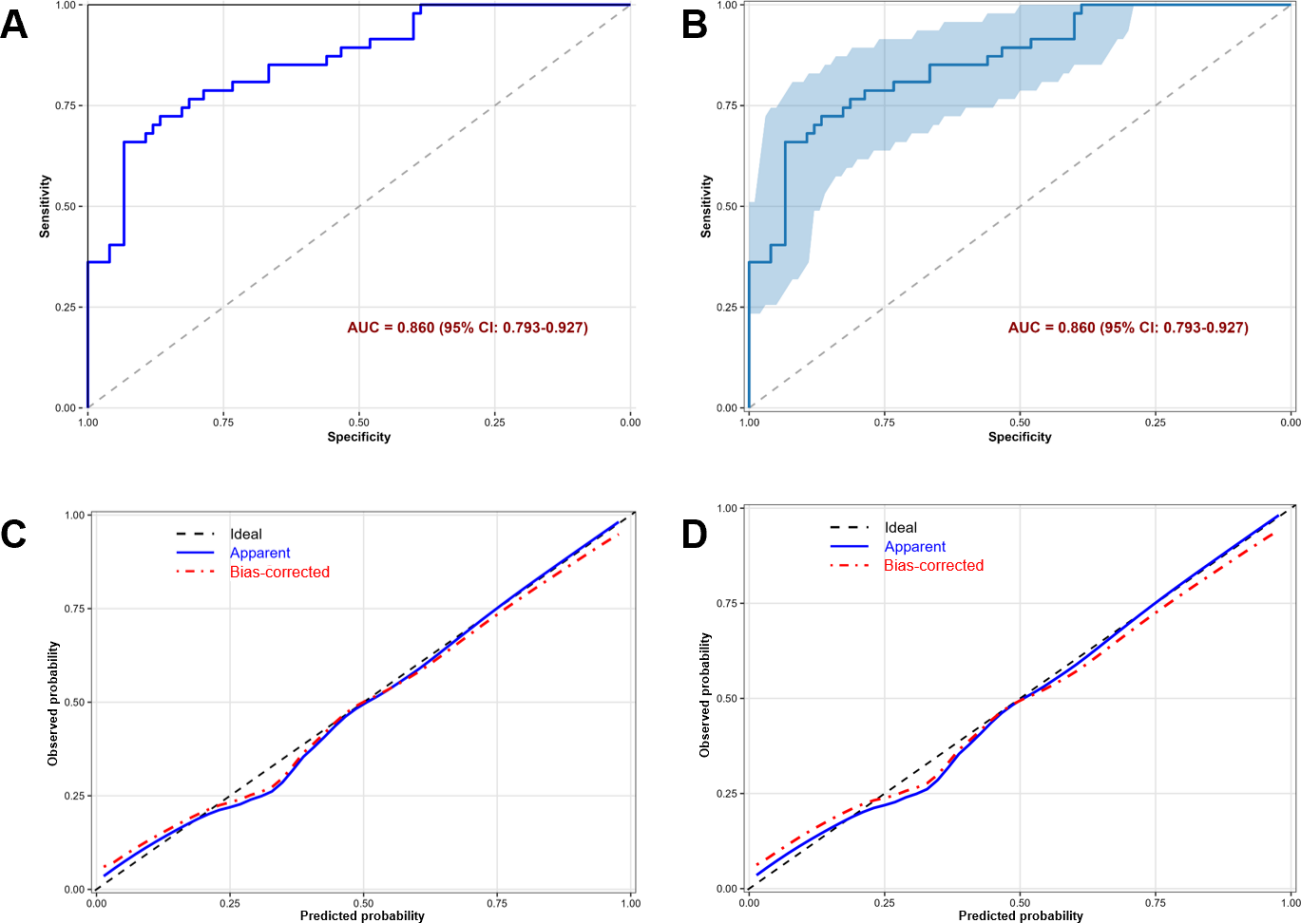
Performance and validation of the nomogram model. (A) The model achieved a concordance index (C-index) of 0.860 (95% CI 0.793‐0.927). (B) Internal validation with 1000 bootstrap resamples yielded a calibrated C-index of 0.860. (C) Calibration was assessed via a calibration plot comprising 3 characteristic curves: the apparent curve representing raw model predictions, the bias-corrected curve reflecting bias-adjusted predictions, and the ideal curve denoting perfect prediction; both the raw and bias-corrected curves demonstrated close alignment with the ideal trajectory, confirming satisfactory model calibration. (D) Calibration curve with internal validation. AUC: area under the receiver operating characteristic curve.

The discriminative ability of the PSCI risk prediction model was evaluated using the concordance index (C-index), which is mathematically equivalent to the AUC. The model achieved a C-index of 0.860 (95% CI 0.793‐0.927, sensitivity=93.3%, and specificity=66.0%; Figure 5A). Internal validation with 1000 bootstrap resamples yielded a calibrated C-index of 0.860. Both values significantly exceeded the reference threshold of 0.75, indicating robust discriminative performance (Figure 5B).

Calibration was assessed via a calibration plot comprising 3 characteristic curves: the apparent curve representing raw model predictions, the bias-corrected curve reflecting bias-adjusted predictions, and the ideal curve denoting perfect prediction. Both the raw and bias-corrected curves demonstrated close alignment with the ideal trajectory, confirming satisfactory model calibration (Figure 5C). The apparent performance line, bias-corrected performance line, and ideal line also show close alignment during internal model validation using bootstrap resampling (Figure 5D).

### Comparative Analysis of Neuroimaging Metrics in PSCI vs NPSCI Cohorts

The analysis of admission brain MRI scans revealed a significantly higher prevalence of frontal lobe infarcts (16/47, 34% vs 11/75, 15%; χ^2^_1_*=*6.29, *P*=.01), temporal lobe involvement (13/47, 28% vs 6/75, 8%; χ^2^_1_=8.49, *P*=.004), larger infarct volumes (*χ^2^*_2_=11.27, *P*=.004), and more severe white matter hyperintensities (WMHs; Fazekas grade ≥2: 22/47, 47% vs 15/75, 20%; χ^2^_2_=10.00, *P*=.007) in patients with PSCI vs NPSCI (Table 5). No significant intergroup differences (*P*>.05) were observed in anterior/posterior circulation distribution or moderate-to-severe large vessel stenosis/occlusion (see Table 5 for exact *P* values). These findings implicate frontal-temporal lesions and white matter disruption as neuroanatomical substrates for PSCI.

**Table 5.** Comparative analysis of neuroimaging parameters between poststroke cognitive impairment (PSCI) and non–poststroke cognitive impairment (NPSCI) cohorts.

Variable	PSCI (n=47), n (%)	NPSCI (n=75), n (%)	χ^2^ (*df*)	*P* value
Stroke infarct location				
Frontal lobe	16 (34)	11 (15)	6.29 (1)	.01
Parietal lobe	9 (19)	6 (8)	3.33 (1)	.07
Temporal lobe	13 (28)	6 (8)	8.49 (1)	.004
Occipital lobe	8 (17)	9 (12)	0.61 (1)	.44
Internal capsule	2 (4)	3 (4)	—^a^	>.99
Basal ganglia	24 (51)	37 (49)	0.04 (1)	.85
Diencephalon	4 (9)	12 (16)	1.42 (1)	.23
Brainstem	11 (23)	16 (21)	0.07 (1)	.79
Cerebellum	1 (2)	3 (4)	—	>.99
Anterior/posterior circulation				
Anterior circulation	34 (72)	44 (59)	2.34 (1)	.13
Posterior circulation	16 (34)	31 (41)	0.65 (1)	.42
Stroke infarct size	—	—	11.27 (2)	.004
Lacunar infarction (<1.5 cm)	25 (53)	58 (78)	—	—
Small subcortical infarction (1.5-3 cm)	9 (19)	12 (16)	—	—
Large territorial infarction (>3 cm)	13 (28)	5 (7)	—	—
Vascular stenosis (≥50%)	17 (36)	18 (24)	2.09 (1)	.15
WMH^b^	—	—	10.00 (2)	.007
Grade 0 (absent)	13 (28)	28 (37)	—	—
Grade I (punctate foci)	12 (26)	32 (43)	—	—
Grade≥II (early confluent or confluent foci)	22 (47)	15 (20)	—	—

a Not applicable.

b WMH: white matter hyperintensity.

### Quantitative Correlation Analysis Between WMHs and Eye-Tracking Parameters

**Figure 6.**
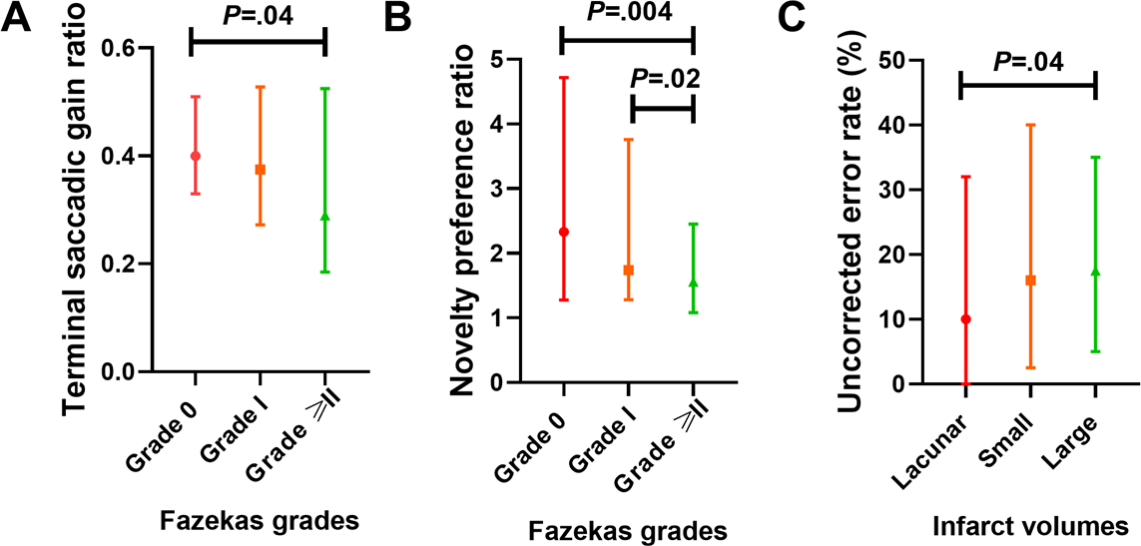
Stratified comparative analysis of eye-tracking metrics by white matter hyperintensity (WMH) severity and infarct volume strata. (A) Comparison of terminal saccadic gain ratio across different WMH severity grades. (B) Comparison of novelty preference ratio across WMH severity grades. (C) Comparison of uncorrected error rate across stroke infarct volume strata.

These stratified analyses were exploratory and not prespecified. Stratified by Fazekas grades on admission brain MRI, the 122 patients with cerebral infarction were categorized into grade 0 (n=41), grade I (n=44), and grade≥II (n=37) cohorts. Significant intergroup differences emerged in terminal saccadic gain ratio (*H*=6.35, *P*=.04) and novelty preference ratio (*H*=11.78, *P*=.003), while other oculomotor metrics showed no statistical significance (all *P*>.05; see Multimedia Appendix 3 for exact *P* values). Post hoc analyses revealed that for terminal saccadic gain ratio, grade≥II demonstrated a 33% reduction vs grade 0 (0.27, IQR 0.17-0.49 vs 0.40 IQR 0.33-0.51; *Z*=19.94, *P*=.04; Figure 6A), but no differences between grade≥II vs I (*Z*=13.20, *P*=.28) or grade I vs 0 (*Z*=6.74, *P*>.99). For novelty preference ratio, grade≥II showed 39% and 18% reductions vs grade 0 (1.43, IQR 1.00-1.63 vs 2.33, IQR 1.27-4.71; *Z*=25.57, *P*=.004; Figure 6B) and grade I (1.43, IQR 1.00-1.63 vs 1.74, IQR 1.28-3.76; *Z*=21.88, *P*=.02), respectively, with no grade 0-I difference (*Z*=3.69, *P*>.99). This dose-dependent impairment pattern indicates that severe WMHs (Fazekas≥II) selectively disrupt visuospatial calibration and novelty detection, implicating compromised comprehension and executive function and significant memory decline.

### Quantitative Correlation Analysis Between Infarct Volumes and Eye-Tracking Parameters

These stratified analyses were exploratory and not prespecified. Stratified by cerebral infarct volume on admission MRI, the 122 patients were categorized into lacunar infarction (<1.5 cm, n=83), small infarction (1.5‐3 cm, n=21), and large infarction (>3 cm, n=18) cohorts. Statistical analysis revealed significant intergroup differences exclusively in uncorrected saccadic error rate (*H*=6.22, *P*=.045; Figure 6C), while other oculomotor metrics showed no significance (all *P*>.05; see Multimedia Appendix 4 for exact *P* values). Post hoc comparisons demonstrated patients with large infarction exhibited 18.0% higher uncorrected error rates vs lacunar infarction (28.00, IQR 15.00-44.00 vs 10.00, IQR 0.00-32.00; *Z*=−22.21, *P*=.04), but there were no significant differences between large vs small infarction (*Z*=−13.90, *P*=.65) or small vs lacunar infarction (*Z*=−8.30, *P*>.99). The dose-dependent trend (large>small>lacunar) further suggests that infarct volume thresholds may dictate cognitive-motor integration failure.

## Discussion

### Principal Findings

PSCI represents a prevalent disabling sequela of stroke, characterized by an insidious onset that frequently delays diagnosis until 3 to 6 months post event. This diagnostic window highlights the urgent need for early detection tools, as untreated PSCI leads to irreversible cognitive decline, severely impacting quality of life and imposing substantial health care system burdens [34]. Current diagnostic limitations such as neuroimaging costs, psychometric scale inaccuracies in patients with aphasic/hemiparetic, and delayed assessment prompted our development of an accessible eye-tracking predictive model.

Among 122 patients with acute ischemic stroke, we observed a 3-month PSCI incidence of 38.5% (47/122), consistent with established epidemiological data [35]. Multivariable regression analysis identified key independent predictors of PSCI: demographic factors, including female sex, advanced age, and lower education level; clinical indicators of higher admission NIHSS score and elevated mRS score; and the oculomotor biomarker of prolonged correct saccade latency. Significantly, eye-tracking biomarkers differentiated PSCI from patients with NPSCI, demonstrating increased saccade latencies and error rates alongside decreased peak velocity, terminal saccadic gain, and novelty preference ratio, while patients with NPSCI exhibited normative oculomotor profiles indistinguishable from HCs. This specificity supports the validity of our integrated nomogram as a rapid, cost-effective tool for acute-phase PSCI risk stratification, facilitating timely interventions during the period of peak neuroplasticity.

### Prediction Model for PSCI Combining AI Eye Tracking and Clinical Demographics

Advanced age and lower educational attainment represent nonmodifiable risk factors for PSCI, consistent with established epidemiological evidence [36,37]. A multicenter cross-sectional study demonstrated escalating PSCI incidence with age, demonstrating 1.887-fold elevated risk in patients ≥75 years compared to younger cohorts [1]. Furthermore, PSCI prevalence exhibits a socioeconomic gradient, disproportionately affecting individuals with lower education and income. The neurobiological mechanisms of education-cognition relationships are explained by 2 conceptual frameworks: the cognitive reserve hypothesis posits that advanced education enhances neural resilience against age-related decline, while the compensatory plasticity model suggests that higher education enables functional reorganization through preserved cognitive domains compensating for impaired functions [38].

NIHSS and mRS scores, which are validated measures of stroke severity, constitute robust predictors of long-term functional outcomes. Higher scores correlate with increased disability and mortality and, specifically, predict the development of PSCI (adjusted OR 1.56 per NIHSS point increase in our cohort) [39]. Pathophysiologically, elevated NIHSS scores reflect more extensive neuroanatomical damage, heightening vulnerability to cognitive domain impairment.

Although female patients showed higher PSCI prevalence in our cohort (18/47, 38% vs 14/75, 19% in NPSCI), it was not an independent predictor in multivariate modeling. This observation aligns with reports of poorer poststroke functional outcomes in women, including greater activity limitations, reduced quality of life, and higher depression rates [40,41]. Notably, while PSCI incidence is compared between sexes, cognitive phenotypes differ: female patients demonstrate predominant deficits in attention, executive function, and language domains, while male patients display greater impairment in verbal memory [40]. Screening tool performance also varies by sex, with MMSE showing higher sensitivity but lower specificity for PSCI detection in women and MoCA exhibiting sex-dependent psychometric properties. These findings necessitate sex-stratified interpretation of cognitive screening instruments to reduce diagnostic inaccuracies.

Strong neuroanatomical connections exist between higher-order cognition and core oculomotor systems. Abstract cognitive tasks engage oculomotor circuits in primates, as manifested through cognitively modulated microsaccades [42]. Observed disparities in eye-tracking metrics between PSCI and NPSCI cohorts reflect multidimensional cognitive dysfunction encompassing attentional control, memory consolidation, and executive processing. Within the antisaccade paradigm, saccadic latency, the interval between stimulus onset and saccade initiation, reflects premotor decision-making processes. Primate neurophysiological studies confirm that saccade programming activates decision-related neurons in frontal eye fields and superior colliculi, wherein saccadic accuracy correlates with neuronal discharge patterns in oculomotor-integration zones [43-45]. Parameters, including latency, amplitude, peak velocity, and positional error, provide quantifiable proxies for decision accuracy and temporal processing [45].

Compared to prosaccades, antisaccades require heightened attentional and executive resources. This task recruits the dorsolateral prefrontal cortex, anterior cingulate cortex, supplementary eye field, and parietal eye field, with increased frontal activation correlating with enhanced antisaccade performance [28]. In our cohort, prolonged saccadic latency in patients with PSCI indicates impaired executive-attentional network function, consistent with frontoparietal dysfunction delaying stimulus-response integration [12,46,47]. Crucially, corrected saccade latency, requiring directional error correction, was identified as an independent PSCI predictor, indicating sensitivity to disrupted feedback monitoring in prefrontal-basal ganglia circuits [48,49]. During the VPC task, reduced novelty preference ratio in patients with PSCI indicated hippocampal-dependent memory impairment. Patients with NPSCI exhibited preserved familiarity discrimination, whereas patients with PSCI allocated equivalent dwell times to novel and familiar images, suggesting declarative memory consolidation failure. Notably, patients with NPSCI demonstrated normative eye-tracking profiles indistinguishable from HCs, confirming stroke-specific cognitive pathology rather than global oculomotor disruption. These oculomotor signatures provide direct windows into disrupted cortical-subcortical circuitry in PSCI.

Current evidence supports the diagnostic utility of eye tracking across neurodegenerative disorders: AD (AUC=0.91) [12], Parkinson dementia, and schizophrenia. Our integrated PSCI prediction model demonstrated comparable discriminative capacity (AUC=0.86, sensitivity=93.3%, and specificity=66.0%). Independent predictors included advanced age, lower education, higher NIHSS scores, and prolonged correct saccade latency, establishing this framework as a rapid biomarker-based stratification tool.

### Correlations Between Neuroimaging Markers and Quantitative Eye-Tracking Metrics

These behavioral patterns implicate underlying neural substrates, which were validated through structural neuroimaging analyses. Neuroimaging analysis confirmed significant associations between PSCI and frontal/temporal lobe infarcts, larger infarct volumes, and severe WMH (Fazekas grade ≥ II). Frontal lesions disrupt attention-executive networks [50], while temporal infarcts impair hippocampal memory consolidation and language processing [35,51]. Moderate-to-severe WMH are established risk factors for PSCI [52,53], likely through the disruption of neuronal networks subserving cognitive reserve. Notably, left anterior thalamic radiation WMH specifically correlates with attention/executive dysfunction, whereas occipital callosal radiation lesions predominantly affect processing speed [54]. Larger infarct volumes predict greater cognitive vulnerability through extensive neocortical involvement. Thus, infarction location/size and WMH severity collectively contribute to cognitive decline.

These oculomotor biomarkers reflect underlying neuroimaging pathology. Significant oculomotor correlates were identified: WMH severity is inversely associated with terminal saccadic gain and novelty preference ratios, while infarct volume is positively correlated with uncorrected saccadic error rates (significantly higher in large vs lacunar infarcts). Mechanistically, reduced terminal gain indicates visuospatial-executive impairment; diminished novelty preference reflects declarative memory deficits; and elevated uncorrected errors signify attentional monitoring dysfunction [45]. These findings establish oculomotor metrics as structural pathology surrogates. Reduced terminal gain/novelty preference indicates severe WMH, while elevated uncorrected errors associate with large infarct volume. For patients contraindicated for neuroimaging, eye tracking provides a clinically feasible alternative for domain-specific cognitive assessment.

Implementing accessible methodologies for early cognitive impairment prediction is paramount in stroke care. Eye-tracking technology offers distinct clinical advantages by integrating audiovisual task instructions, including on-screen text, subtitles, and voice-over narration, to ensure accessibility across varying levels of literacy and physical ability. By relying on gaze-based responses, the method eliminates the need for verbal or manual interaction, making it particularly suitable for patients with aphasia or dominant-hand paresis. As a mobile screening tool, it enables efficient cognitive phenotyping through quantitative oculomotor biomarkers, delivering actionable clinical insights promptly.

Furthermore, integrated within mHealth platforms, tablet-based eye tracking supports scalable, low-cost screening beyond traditional clinical settings. This approach facilitates real-time data acquisition and seamless telehealth integration, promoting equitable access to follow-up care—especially for individuals in remote or underserved regions. Beyond accelerating clinical decision-making, this mHealth-enabled paradigm allows decentralized longitudinal monitoring of disease progression and treatment response, including remotely administered home assessments.

This study pioneers the multidisciplinary integration of eye-tracking analytics with clinical demographics to develop a predictive model for PSCI. We established neurobiological correlations between oculomotor signatures and structural neuroimaging markers, thereby expanding the repertoire of neurobehavioral biomarkers while offering a cost-effective framework for stratifying post-stroke cognitive risk.

### Limitations

Several limitations of this study should be acknowledged. First, the sample size was relatively modest (122 patients with complete follow-up, along with 20 HCs) and derived from a single center, which may limit statistical power and generalizability of the findings. Future multicenter studies with larger cohorts are needed to validate and refine our predictive model. Second, the follow-up period was limited to 3 months, which may not capture later-onset cognitive decline or the long-term trajectory of PSCI. Extended follow-up (eg, 6 or 12 mo) would provide a more comprehensive understanding of the evolution of cognitive impairment and the stability of eye-tracking biomarkers. Third, as an observational diagnostic study with a relatively small sample size, our findings should be considered exploratory rather than confirmatory. Prospective randomized controlled trials with adequate sample size and appropriate blinding are required to further validate the diagnostic sensitivity and specificity of this tablet-based eye-tracking tool, particularly if it is to be considered as a software-as-a-medical-device for regulatory or clinical registration purposes. Fourth, the oculomotor battery was restricted to VPC and antisaccade tasks. Although these tasks are well-established for assessing executive function and memory, incorporating additional paradigms such as smooth pursuit, free viewing, or memory-guided saccades could offer a more complete assessment of the spectrum of cognitive deficits in PSCI. Fifth, our study focused exclusively on acute cerebral infarction; therefore, the findings may not directly generalize to other stroke subtypes (eg, hemorrhagic stroke) or to diverse ethnic and demographic populations. Validation across different stroke types and settings is warranted. Sixth, while we correlated eye-tracking metrics with WMH severity and infarct volume, we did not integrate more advanced neuroimaging biomarkers (eg, diffusion tensor imaging or resting-state functional MRI). Future studies linking oculomotor impairments to structural and functional connectivity measures would further strengthen the neurobiological basis of our findings. Seventh, the study employed a purposive (nonprobability) sampling strategy, which may introduce selection bias and limit the representativeness of the sample. Eighth, there was heterogeneity in acute stroke treatments among participants (eg, intravenous thrombolysis and endovascular thrombectomy), which could influence cognitive outcomes; although we adjusted for baseline NIHSS and mRS scores, residual confounding cannot be excluded.

### Conclusions

Age, educational level, admission NIHSS score, and correct saccade latency were identified as independent predictors of 3-month PSCI and were incorporated into a nomogram model. The model demonstrated robust performance, achieving high predictive accuracy (AUC=0.86), strong discriminative capacity, and clinically useful interpretability. This tool demonstrates potential for early identification of patients with high-risk PSCI, thereby enabling timely and personalized interventions. Its integration into standard clinical pathways—particularly through mHealth platforms—may significantly enhance strategic decision-making and improve long-term outcomes for vulnerable populations.

## Supplementary material

10.2196/83194 Multimedia Appendix 1 Comparative analysis of eye-tracking metrics between poststroke cognitive impairment and non–poststroke cognitive impairment cohorts.

10.2196/83194 Multimedia Appendix 2 Comparative analysis of eye-tracking metrics between healthy control and non–poststroke cognitive impairment cohorts.

10.2196/83194 Multimedia Appendix 3 Analysis of eye-tracking metrics by white matter hyperintensity severity grade.

10.2196/83194 Multimedia Appendix 4 Analysis of eye-tracking metrics by infarct volume strata.

10.2196/83194 Checklist 1 STARD-2015 checklist.

## Abbreviations

AD: Alzheimer disease
AUC: area under the receiver operating characteristic curve
C-index: concordance index
HC: healthy control
MCI: mild cognitive impairment
mHealth: mobile health
MMSE: Mini-Mental State Examination
MoCA: Montreal Cognitive Assessment
MRI: magnetic resonance imaging
mRS: Modified Rankin Scale
NIHSS: National Institutes of Health Stroke Scale
NPSCI: non–poststroke cognitive impairment
OR: odds ratio
PSCI: poststroke cognitive impairment
VPC: visual paired comparisons
WMH: white matter hyperintensity
