# Supplementary material for: Utility of Tablet-Based Eye Tracking for Early Screening of Poststroke Cognitive Impairment: Diagnostic Cohort Study
Source: JMIR Mhealth Uhealth. 2026 Aug 6;14:e83194. doi: 10.2196/83194 (PMC13446795; doi:10.2196/83194)
Supplement: Supplementary file 1 [file mhealth-v14-e83194-s001.docx]

**Appendix 1. Comparative analysis of eye-tracking metrics between PSCI and NPSCI cohorts**

| Variable | PSCI(*n*=47) | NPSCI(*n*=75) | *t/Z* | *P* value |
| --- | --- | --- | --- | --- |
| Latency(ms) | 339.84(245.13,539.69) | 182.17(134.17,284.01) | -4.77 | <.001 |
| Correct Saccade Latency(ms) | 322.96(209.45, 445.59) | 194.55(141.50, 299.75) | -4.03 | <.001 |
| Error Rate (%) | 56.45±14.929 | 54.67±12.610 | -0.71 | .48 |
| Peak Velocity (Horizontal, pixel/ms) | 3.86(2.81, 5.20) | 4.40(3.42, 5.50) | -2.01 | .04 |
| Correction Latency(ms) | 179.98(127.76,325.89) | 170.82(99.99,254.52) | -0.94 | .35 |
| Uncorrected Error Rate (%) | 30.00(15.00, 42.00) | 5.00(0.00, 28.00) | -4.24 | <.001 |
| Corrected Saccade Latency(ms) | 400.24(302.63, 612.31) | 266.63(214.98, 360.13) | -4.62 | <.001 |
| Pre-saccadic Fixation Count(count) | 4.25(2.92,6.05) | 4.25(3.05, 5.50) | -0.13 | .90 |
| Post-saccadic Fixation Count(count) | 14.85(10.85,19.30) | 17.78(12.60,22.67) | -2.20 | .03 |
| Fixation Stability(count) | 1.85(1.50,2.25) | 1.95(1.70,2.25) | -1.21 | .23 |
| Blinks During Fixation(count) | 0.00(0.00, 0.00) | 0.00 (0.00, 0.00) | -0.18 | .86 |
| Initial Saccadic Gain(pixel) | 7.10(4.67, 16.29) | 6.44(2.64, 13.15) | -1.14 | .26 |
| Terminal Saccadic Gain Ratio | 0.27(0.17, 0.48) | 0.41(0.31, 0.53) | -2.97 | .003 |
| Novelty Preference Ratio | 1.44(0.97, 1.70) | 2.12(1.27, 4.56) | -3.44 | .001 |
| Total Fixation Duration (ms) | 3589.64(2926.37,4116.25) | 3536.29(2746.40,3826.29) | -1.09 | .26 |
| Fixation Events with Blinks(count) | 0.00(0.00, 0.10) | 0.00(0.00, 0.10) | -0.57 | .57 |
