# Supplementary material for: Utility of Tablet-Based Eye Tracking for Early Screening of Poststroke Cognitive Impairment: Diagnostic Cohort Study
Source: JMIR Mhealth Uhealth. 2026 Aug 6;14:e83194. doi: 10.2196/83194 (PMC13446795; doi:10.2196/83194)
Supplement: Supplementary file 2 [file mhealth-v14-e83194-s002.docx]

**Appendix 2. Comparative analysis of eye-tracking metrics between HC and NPSCI cohorts**

| Variable | HC(*n*=20) | NPSCI(*n*=75) | *t/Z* | *P* value |
| --- | --- | --- | --- | --- |
| Latency(ms) | 197.43(146.91, 27.00) | 182.17(134.17,284.01) | -0.59 | .58 |
| Correct Saccade Latency(ms) | 225.65(147.17,293,79) | 194.55(141.50, 299.75) | -0.51 | .61 |
| Error Rate(%) | 48.45±12.22 | 54.67±12.610 | 1.97 | .052 |
| Peak Velocity (Horizontal, pixel/ms) | 4.13(2.79,6.55) | 4.40(3.42, 5.50) | -0.46 | .65 |
| Correction Latency(ms) | 104.75(76.03,207.84) | 170.82(99.99,254.52) | -1.52 | .13 |
| Uncorrected Error Rate(%) | 5.50(0.00,26.00) | 5.00(0.00, 28.00) | -0.11 | .91 |
| Corrected Saccade Latency(ms) | 254.50(217.79,349.64) | 266.63(214.98, 360.13) | -0.39 | .70 |
| Pre-saccadic Fixation Count(count) | 4.23(2.47,6.16) | 4.25(3.05, 5.50) | -0.72 | .47 |
| Post-saccadic Fixation Count(count) | 17.71(12.44,22.98) | 17.78(12.60,22.67) | -0.43 | .67 |
| Fixation Stability(count) | 2.00(1.59,2.32) | 1.95(1.70,2.25) | -0.24 | .81 |
| Blinks During Fixation(count) | 0.00(0.00,0.09) | 0.00 (0.00, 0.00) | -0.71 | .48 |
| Initial Saccadic Gain(pixel) | 4.51(2.97,12.13) | 6.44(2.64, 13.15) | -0.31 | .76 |
| Terminal Saccadic Gain Ratio | 0.35(0.23,0.60) | 0.41(0.31, 0.53) | -0.73 | .47 |
| Novelty Preference Ratio | 2.28(1.78,4.80) | 2.12(1.27, 4.56) | -0.98 | .33 |
| Total Fixation Duration (ms) | 3571.31(3148.85, 3777.12) | 3536.29(2746.40, 3826.29) | -0.50 | .62 |
| Fixation Events with Blinks(count) | 0.00(0.00,0.00) | 0.00(0.00, 0.10) | -0.24 | .81 |
