# Supplementary material for: Utility of Tablet-Based Eye Tracking for Early Screening of Poststroke Cognitive Impairment: Diagnostic Cohort Study
Source: JMIR Mhealth Uhealth. 2026 Aug 6;14:e83194. doi: 10.2196/83194 (PMC13446795; doi:10.2196/83194)
Supplement: Supplementary file 3 [file mhealth-v14-e83194-s003.docx]

**Appendix Table 3. Analysis of Eye-Tracking Metrics by WMH Severity Grade**

**Appendix Table 3.1. Comparative analysis of eye-tracking metrics across WMH severity grades**

| Variable | Grade 0 (*n*=41) | Grade I (*n*=44) | Grade≥II (*n*=37) | *P* value |
| --- | --- | --- | --- | --- |
| Latency(ms) | 193.83(155.95,400.08) | 263.17(155.28,354.41) | 290.60(150.59,455.72) | .60 |
| Correct Saccade Latency(ms) | 221.60(154.64,374.78) | 264.67(173.03,333.07) | 252.49(155.02,387.36) | .98 |
| Error Rate(%) | 54.98±15.584 | 54.55±10.855 | 56.73±14.165 | .75 |
| Peak Velocity (Horizontal, pixel/ms) | 4.49(3.73,5.50) | 4.09(3.26,5.53) | 3.86(2.88,5.09) | .31 |
| Correction Latency(ms) | 140.72(106.05,237.47) | 197.89(114.15,259.00) | 187.48(109.92,285.26) | .35 |
| Uncorrected Error Rate(%) | 10.00(0.00,34.00) | 15.50(0.00,35.00) | 20.00(5.00,41.00) | .21 |
| Corrected Saccade Latency(ms) | 281.43(221.04,393.05) | 328.69(229.33,431.64) | 345.64(242.77,522.51) | .51 |
| Pre-saccadic Fixation Count(count) | 4.35(2.95,5.39) | 4.03(3.02,6.16) | 4.35 (3.13,6.22) | .90 |
| Post-saccadic Fixation Count(count) | 17.18(12.51,21.30) | 17.70(12.40,21.35) | 16.45(10.66,21.30) | .68 |
| Fixation Stability(count) | 1.90(1.60,2.15) | 2.20(1.71,2.40) | 1.95(1.59,2.25) | .34 |
| Blinks During Fixation(count) | 0.00(0.00,0.00) | 0.00(0.00,0.05) | 0.00(0.00,0.06) | .50 |
| Initial Saccadic Gain(pixel) | 6.19(2.28,14.60) | 7.28(3.60,12.61) | 7.84 (3.42,20.35) | .31 |
| Terminal Saccadic Gain Ratio | 0.40(0.33,0.51) | 0.37(0.27,0.52) | 0.27(0.17,0.49) | .04 |
| Novelty Preference Ratio | 2.33(1.27,4.71) | 1.74(1.28,3.76) | 1.43(1.00,1.63) | .003 |
| Total Fixation Duration (ms) | 3616.29(2934.71,3837.95) | 3576.30(2834.43,3972.81) | 3509.65(1187.27,4006.26) | .94 |
| Fixation Events with Blinks(count) | 0.00(0.00,0.10) | 0.00(0.00,0.10) | 0.00(0.00,0.00) | .60 |

**Appendix Table 3.2. Comparison of terminal saccadic gain ratio across different WMH severity grades**

|  | Grade 0 (*n*=41) | Grade I (*n*=44) | Grade≥II (*n*=37) | *H/Z* | *P* value |
| --- | --- | --- | --- | --- | --- |
| Terminal Saccadic Gain Ratio | 0.40(0.33,0.51) | 0.37(0.27,0.52) | 0.27(0.17,0.49) | 6.35 | .04 |
| Grade≥II - I |  |  |  | 13.20 | .28 |
| Grade≥II - 0 |  |  |  | 19.94 | .04 |
| Grade I - 0 |  |  |  | 6.74 | >0.99 |

**Appendix Table 3.3. Comparison of novelty preference ratio across WMH severity grades**

|  | Grade 0 (*n*=41) | Grade I (*n*=44) | Grade≥II (*n*=37) | *H/Z* | *P* value |
| --- | --- | --- | --- | --- | --- |
| Novelty Preference Ratio | 2.33(1.27,4.71) | 1.74(1.28,3.76) | 1.43(1.00,1.63) | 11.78 | .003 |
| Grade≥II - I |  |  |  | 21.88 | .02 |
| Grade≥II - 0 |  |  |  | 25.57 | .004 |
| Grade I - 0 |  |  |  | 3.69 | >0.99 |
