# Supplementary material for: Utility of Tablet-Based Eye Tracking for Early Screening of Poststroke Cognitive Impairment: Diagnostic Cohort Study
Source: JMIR Mhealth Uhealth. 2026 Aug 6;14:e83194. doi: 10.2196/83194 (PMC13446795; doi:10.2196/83194)
Supplement: Supplementary file 4 [file mhealth-v14-e83194-s004.docx]

**Appendix Table 4. Analysis of Eye-Tracking Metrics by Infarct Volume Strata**

**Appendix Table 4.1. Comparative analysis of eye-tracking metrics across stroke infarct volume strata**

| Variable | Lacunar infarction (<1.5 cm)(*n*=83) | Small subcortical infarction (1.5 cm - 3 cm)(*n*=21) | Large territorial infarction (>3 cm)(*n*=18) | *P* value |
| --- | --- | --- | --- | --- |
| Latency(ms) | 199.52(154.29,322.51) | 253.98(142.07,416.28) | 377.31(225.16,518.08) | .11 |
| Correct Saccade Latency(ms) | 209.45(155.30,305.64) | 282.82(150.55,498.95) | 306.03(186.16,412.24) | .12 |
| Error Rate(%) | 55.08±12.61 | 57.10±16.03 | 54.56±16.038 | .80 |
| Peak Velocity (Horizontal, pixel/ms) | 4.30(3.16,5.42) | 4.40(3.47,5.69) | 3.96(3.07,4.73) | .56 |
| Correction Latency(ms) | 170.82(99.99,254.52) | 185.69(135.40,301.48) | 191.09(130.53,246.84) | .54 |
| Uncorrected Error Rate(%) | 10.00(0.00,32.00) | 16.00(2.50,40.00) | 28.00(15.00,44.25) | .045 |
| Corrected Saccade Latency(ms) | 281.48(218.21,421.18) | 345.64(269.82,546.13) | 383.74(322.23,544.31) | .07 |
| Pre-saccadic Fixation Count(count) | 4.00(2.92,5.29) | 4.90(2.70,6.47) | 5.00(3.82,8.32) | .06 |
| Post-saccadic Fixation Count(count) | 17.00(11.10,20.76) | 17.45(12.21,22.75) | 17.10(13.60,26.28) | .77 |
| Fixation Stability(count) | 1.90(1.61,2.25) | 1.90(1.62,2.20) | 2.07(1.88,2.32) | .38 |
| Blinks During Fixation(count) | 0.00(0.00,0.05) | 0.00(0.00,0.11) | 0.05(0.00,0.10) | .16 |
| Initial Saccadic Gain(pixel) | 7.18(4.67,15.20) | 5.33(2.34,9.82) | 5.55(2.18,16.11) | .27 |
| Terminal Saccadic Gain Ratio | 0.37(0.25,0.51) | 0.33(0.22,0.57) | 0.37(0.14,0.52) | .87 |
| Novelty Preference Ratio | 1.78(1.27,3.76) | 1.43(1.06,2.85) | 1.47(0.83,3.13) | .11 |
| Total Fixation Duration (ms) | 3536.29(2755.28,3949.60) | 3582.98(3223.38,3776.30) | 3817.95(1971.47,4030.43) | .90 |
| Fixation Events with Blinks(count) | 0.00(0.00,0.10) | 0.00(0.00,0.10) | 0.00(0.00,0.10) | .99 |

**Appendix Table 4.2. Comparison of uncorrected error rate across stroke infarct volume strata**

|  | Lacunar infarction (<1.5 cm)(*n*=83) | Small subcortical infarction (1.5 cm - 3 cm)(*n*=21) | Large territorial infarction (>3 cm)(*n*=18) | *H/Z* | *P* value |
| --- | --- | --- | --- | --- | --- |
| Uncorrected Error Rate(%) | 10.00(0.00,32.00) | 16.00 (2.50,40.00) | 28.00(15.00,44.00) | 6.22 | .045 |
| Lacunar infarction - Small subcortical infarction |  |  |  | -8.30 | >0.99 |
| Lacunar infarction - Large territorial infarction |  |  |  | -22.21 | .04 |
| Small subcortical infarction - Large territorial infarction |  |  |  | -13.90 | .65 |
